# Multifactorial intervention for hip and pelvic fracture patients with mild to moderate cognitive impairment: study protocol of a dual-centre randomised controlled trial (OF-CARE)

**DOI:** 10.1186/s12877-019-1133-z

**Published:** 2019-04-30

**Authors:** Anja Dautel, Tobias Eckert, Michaela Gross, Klaus Hauer, Martina Schäufele, André Lacroix, Ingrid Hendlmeier, Bastian Abel, Rebekka Pomiersky, Julia Gugenhan, Gisela Büchele, Katrin C. Reber, Clemens Becker, Klaus Pfeiffer

**Affiliations:** 1 Department of Clinical Gerontology and Geriatric Rehabilitation, Robert-Bosch-Hospital, Auerbachstrasse 110, 70376 Stuttgart, Germany; 2 Agaplesion Bethanien Hospital, Heidelberg, Germany; 3 Hochschule Mannheim, University of Applied Science, Mannheim, Germany; 4 Institute of Epidemiology and Medical Biometry, Ulm University, Ulm, Germany; 5 Department of Health Economics and Health Services Research, University Medical Centre Hamburg, Hamburg, Germany

**Keywords:** RCT, Multifactorial, Home-based, Exercise program, Care counselling, Lay instructor, Elderly, Hip or pelvic fracture, Cognitive impairment, Cost-effectiveness

## Abstract

**Background:**

A hip or pelvic fracture is a major fall-related injury which often causes a decline in mobility performance and physical activity. Over 40% of patients with hip fracture have cognitive impairment or dementia and poorer rehabilitation outcomes than those without cognitive impairment. In this subgroup, there is a lack of evidence on the best practices supporting recovery. The main aim of this study is to investigate the effects of a transitional care intervention after inpatient rehabilitation on physical activity and functional performance in this group of cognitively impaired patients.

**Methods/design:**

This dual-centre, randomised controlled trial compares a multifactorial intervention with usual care as control condition. Two hundred and forty community-dwellers (≥ 65 years) with a hip or pelvic fracture and mild to moderate cognitive impairment (MMSE 17–26) are recruited at the end of inpatient rehabilitation.

The four-month intervention consists of (a) an individually tailored, progressive home exercise program and physical activity promotion delivered by professional instructors and lay instructors (two home visits per week) and (b) a long-term care counselling approach addressing unmet care needs, pleasurable activities, and caregiver issues if needed. Primary outcome parameters are physical activity, measured as daily walking duration with an accelerometer-based activity monitor (activPAL™) over 72 h, and functional performance, assessed with Short Physical Performance Battery sum scores. Secondary outcome parameters are fear of falling, fall related self-efficacy, falls, quality of life, depression and activity of daily living. Data are collected at the end of rehabilitation, before the intervention at the patient’s home (baseline), after four months (post-intervention), and seven months (follow-up). In addition to completer and intent-to-treat analyses of outcomes, economic data and incremental cost-effectiveness are analysed.

**Discussion:**

Existing service models of volunteer services and legal counselling provided by care counsellors were considered when developing the intervention protocol. Therefore, it should be feasible to translate and deliver the intervention into real-world practice if it has been demonstrated to be effective.

**Trial registration:**

German Clinical Trials Register, DRKS00008863 (Accessed 17 Apr 2019), ISRCTN registry, ISRCTN69957256 (Accessed 17 Apr 2019).

## Background

The high incidence of fall related hip and pelvic fractures as well as the associated negative consequences have been widely described in the literature [1–4]. Mortality rates after hip-surgery are 5 to 10% within 30 days and about 33% after one year [5]. Within six months after fracture, only 40 to 70% regain their prior independence for basic activities of daily living (ADLs) [1]. Between 10 to 20% of hip fracture patients are dependent on long-term care and institutionalised as a consequence of the fracture [1]. Thus, high socioeconomic burden beyond acute care and rehabilitation is apparent [6]. Demographic changes in the future indicate further increases in health and social costs for this patient group [7].

Previous research has identified several predictors for reduced or delayed functional recovery after hip fractures. This includes high age, low pre-fracture level of functioning, reduced walking ability, high prevalence of comorbidities and certain fracture types (e.g. inter−/subtrochanteric versus cervical) [8, 9]. Other factors are fear of falling [10], depression [11, 12] and cognitive impairment [13, 14].

Over 40% of all patients with fall-related hip fracture are cognitively impaired [15]. These patients show poorer mobility outcomes and higher ADL dependence one year after the fracture [13, 16], and in consequence a significantly increased risk of nursing home admission [13].

Although, there is no gold standard intervention for improving mobility after hip fracture [17], structured exercises with appropriate progression and intensity have been found to be effective [18, 19]. While supervised exercise regimes can be implemented easily during inpatient rehabilitation, the continuation of regular, progressive and enjoyable exercise regimes after discharge is challenging. Many people, in particular those with cognitive impairment may prefer to exercise at home rather than attending group sessions. While home based exercise interventions with cognitive behavioural components and minimal supervision can improve physical performance of hip fracture patients without cognitive impairment [20], there is only little evidence derived from subgroup analyses or non-randomised studies for cognitively impaired hip fracture patients. These previous results support that rehabilitation appears to be beneficial for this subgroup, in particular if it is tailored to preferences of the patients in interdisciplinary approaches [21]. However, specific interventions for cognitively impaired patients in the transition from hospital to home which address mobility issues and the need for added support and resources (also for their caregivers) are missing from the literature so far [22].

In contrast to current intervention research for hip fracture patients, comparable research for pelvic fracture patients is lacking. Despite the differences in the (non-) operative treatment of the different fracture types, it is assumed that in early discharge and community rehabilitation both patient groups can be addressed with the same intervention approach.

### Objectives

Primary objective of this study is to compare the effect of a multifactorial home program with usual care on physical activity and functional performance (primary outcomes) in hip and pelvic fracture patients with mild to moderate cognitive impairment. Major components of the post-discharge intervention over four months are supervised exercises, physical activity promotion and long-term care counselling. To increase cost effectiveness and the chances for sustainable implementation, lay instructors deliver a part of the intervention.

Secondary objectives are to evaluate the effect of the intervention on fear of falling, fall related self-efficacy, falls, depressive symptoms, quality of life, and activities of daily living. Economic data and incremental cost-effectiveness will be analysed.

Family caregivers, if existing and consenting to participate, received a specific caregiver counselling and are evaluated in addition to the patients.

## Methods and design

**Fig. 1:**
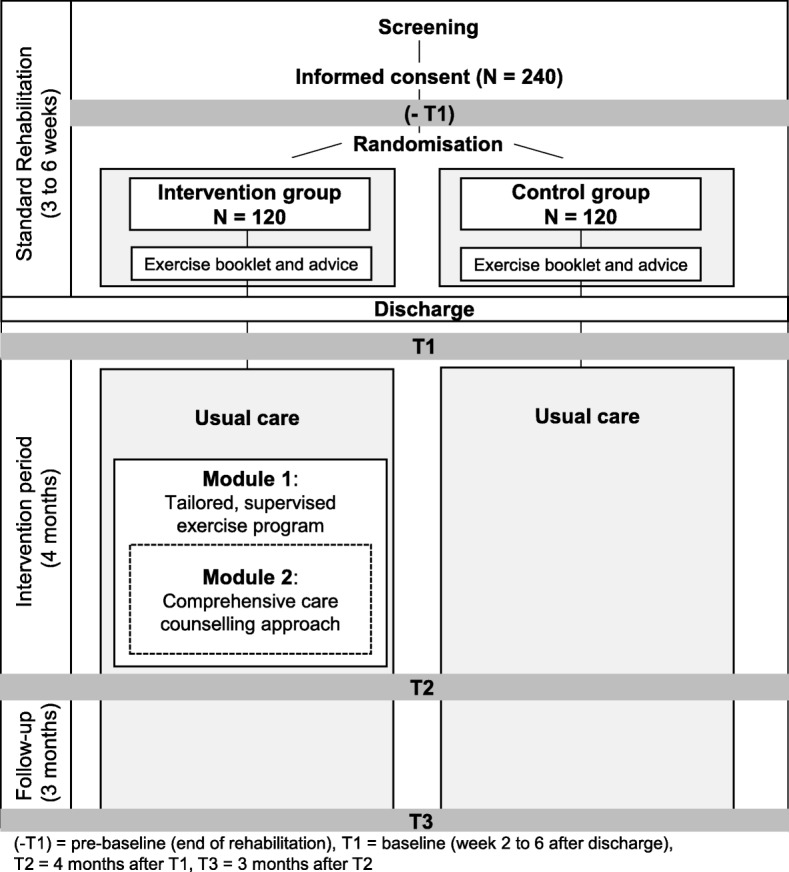
Study design

This study is a dual-centre, randomised controlled trial with blinded assessors. Participants (*N* = 240) were randomly assigned to one of two groups (multifactorial intervention or usual care control group) in a 1:1 ratio after initial assessment (−T1) and before discharge from inpatient rehabilitation. Computer-generated random allocation was done by an independent randomisation centre (University of Ulm) and sealed envelopes are used. The randomisation of participants was conducted by the interventionists. The study design is outlined in Fig. 1*.*

### Recruitment

All hip and pelvic fracture patients admitted to the geriatric rehabilitation departments of the Robert-Bosch-Hospital Stuttgart and the Agaplesion Bethanien Hospital Heidelberg (both Germany) were assessed for eligibility from July 2015 to February 2018. To include patients with complications after fracture or surgery, a maximum interval of 3 months between fracture event and admission to rehabilitation was allowed. Patients who had completed any orthopaedic rehabilitation during this time interval were not assessed.

After medical clearing, the screening and the recruiting were planned within the first two weeks of inpatient rehabilitation and done by trained physiotherapists and sports scientists. If it was unclear that the participant could return home the final screening and recruitment procedure was postponed to the third week. Close communication was maintained with the patient, caregiver as well as rehab staff, to avoid unexpected discharge management to nursing home.

Inclusion criteria were a hip or pelvic fracture within the last three months, mild to moderate cognitive impairment (Mini-Mental State Examination score of 17–26 [23]), age ≥ 65 years, minimum visual acuity (corrected vision, Snellen fraction > 20/400), living in home environment or assisted living and ability to walk 4 m with or without walking aid. Exclusion criteria were delirium (identified by the Confusion Assessment Method [24]), severe somatic or mental illness, terminal disease, moderate to severe aphasia (except amnestic aphasia) or severe apraxia, insufficient hearing ability for receiving calls or no telephone accessibility, insufficient knowledge of German language, and place of residence outside the Stuttgart and Heidelberg area, respectively.

Positive screened persons were informed in verbal and written form about the study program and asked to give written consent. The whole procedure was conducted in a comprehensible way according to the recommendations of Appelbaum [25]. Existing legal guardians or authorized representatives were involved in the information and consent process in any case, otherwise the closest family member if possible.

Family members who provided care for the fracture patients and met the following inclusion criteria were also invited to participate in the study: providing care on a non-commercial base for at least 1.5 h per day or 10.5 h per week (assistance of ADLs, instrumental ADLs (IADLs), supervision, including journey time to care recipient’s home), age ≥ 18 years and willingness to engage in a counselling session at the patient’s home. Exclusion criteria were current mental illness or cognitive impairment that affected the ability to give informed consent, to understand the requirements of the assessments or to participate in the intervention, insufficient hearing ability for receiving calls, no telephone accessibility, and insufficient knowledge of German language. Caregiver recruitment (only one caregiver per patient) had to be completed at least during baseline assessment (T1) at the participant’s home.

### Intervention

#### Overview

**Fig. 2:**
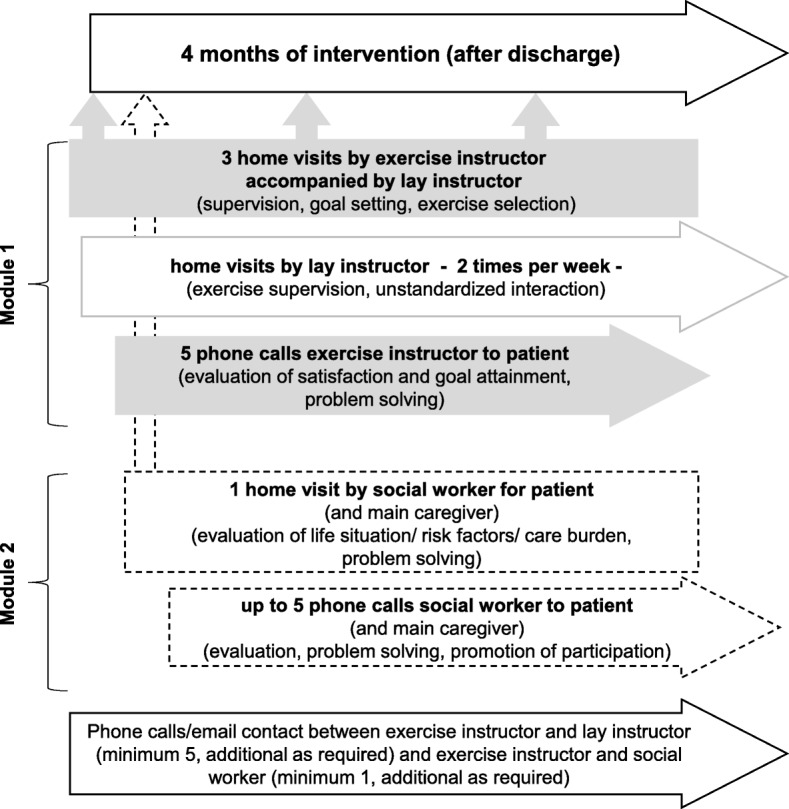
Flow of intervention

The intervention program is outlined according to the TIDierR checklist to ensure a clear and systematic description [26]. The three key components of the multifactorial OF-CARE intervention are physical activity promotion, an individually tailored, progressive, exercise home program (M*odule 1*), and care counselling for the participants and their participating caregivers (if existing) (*Module 2*). Table 1 illustrates the key elements and Fig. 2 the flow of intervention.

**Table 1:** OF-Care key elements of the intervention

Key element	Rationale and brief description
*Structure*	
Interventions starting in the patient’s home early after discharge from inpatient rehabilitation	• Exercise continuity after rehabilitation period (transitional care) • Long-term care counselling addressing new support needs and other adaptation issues at home for both, care recipient and caregiver.
Professional exercise instructor (physiotherapist, sports scientist)	• Appropriate exercise selection and progression to improve strength and balance. • Physical activity promotion and goal setting • Addressing fall and safety risks in the patient’s home and during training • Demonstration of additional self-exercises to patient and family member • Training and supervision of lay instructor
Lay-instructor	• Ensuring exercise continuity through regular home visits • Spending some additional time with enjoyable activities together with the fracture patient (positive reinforcement for the whole program)
Social worker	• Assessment of care needs • Organisation of support if necessary (case management) • Addressing caregiver issues
Linking of intervention to long-term care counselling	• Standardized communication and shared analysis of patient’s needs and activity goals between social worker and exercise instructor
*Contents*	
Individual goal setting	• Application of an iconographical card sorting task to facilitate goal setting in this target group • Enhancing intrinsic motivation and training adherence
Exercise program	• Individually tailored, progressive, supervised exercise regime, 2 times per week (details *see* Table 2) • Additional self-exercises to promote activity on the days without supervised training, 1–4 times per week • (Supervised) physical activities, gradual progression by splitting instrumental activities in single components first
Long-term care counselling	• Structured assessment of needs and wishes of patients with focus on care issues and social participation • Structured problem-solving with the main caregiver (if existing)

The intervention group and the control group both have regular access to standard care during the whole study. Hence, the intervention is an add-on to usual care. To establish a minimum standardization of recommendations given before discharge at both recruitment sites, all participants receive the same face-to-face advice session (max. 60 min) on recommended regular physical exercises and tips for fall prevention. The addressed issues are based on an illustrated advice booklet [27] given to all participants. For those with difficulties in understanding the recommendations, this session can be repeated or, if existing, caregivers are asked to attend.

#### Description of module 1 (physical activities and exercises)

*Module 1* starts with an initial home visit (maximum duration 2 h) two to six weeks post- discharge by an exercise instructor (physiotherapist or sports scientist) together with a lay instructor. This timeframe was chosen according to the experience made during piloting the interventional approach (Gross M, Pfeiffer K: Hip fracture and cognitive impairment: Pilot study of a post-rehabilitation exercise program, unpublished). Participants of this feasibility study often needed a certain time to settle in own home environment after several weeks of acute care and rehabilitation.

Aim of this first visit is (a) to set at least one physical activity goal, (b) to specify a tailored exercise program on strength, balance and gait, and (c) to introduce and instruct the lay instructor.

##### Patient-centred goal setting

Patient-centred goal setting has been shown to contribute to enhanced fall related self-efficacy and balance confidence in patients after hip fracture [28]. A card sorting task with cards of 20 daily physical activities is applied to facilitate the participants in setting their own activity goals. This method is based on visual representation techniques that have been successfully implemented in previous research on caregiving and care planning [29, 30]. The activities displayed on the cards (icon and legend) were derived from existing ADL and IADL assessments and expert interviews when preparing this study. The procedure starts by asking the participants to place the activity cards under heading cards to differentiate between activities they perform with or without the help of others (independent, spouse/child, relatives/friends/neighbours, professional service, unclear, not relevant). The cards relating to activities the participants can do independently and with confidence are put aside. Of the remaining cards, they are asked to select the subjectively most important or suitable activities to improve independence or confidence. Further physical activities not covered by the card set can be added using hand-lettered blank cards. Possible independence and safety goals (e.g. to prevent fall-risk situations) are discussed and prioritized with the participants. Following this, the exercise instructor tries to encourage them to select one to three attainable and meaningful goals. If needed, difficult goals are broken up into easier achievable intermediate goals. Goals with different performance tasks are divided and trained in different subtasks first, if necessary (i.e. climbing stairs, opening heavy front door and carrying the letter mail while walking to achieve the goal “*emptying the letterbox*”). When discussing the implementation of the chosen activities, peers (e.g. caregiver, lay instructor) who are needed for assistance are included.

For recording goal achievement Goal Attainment Scaling [31] is used. This instrument was suggested to be feasible and responsive to change in geriatric rehabilitation settings [32].

##### Specification of tailored exercise program

In the second component of the initial home visit, the exercise instructor (physiotherapists and sports scientists) first discusses with the participants why regular exercise is important (e.g. to attain own physical activity goals, to prevent further falls, to maintain autonomy). An individually tailored training schedule is compiled from a set of balance (standing, weight shifts, walking) and strength (chair rising) exercises with different intensities. For setting the exercise plan, tasks for each exercise component are performed from easy to difficult (based on the participants´ functional capacity and according to the exercise instructor’s assessment). The training protocol is outlined in Table 2. The exercise instructor chooses the tasks the participants are barely able to perform safely with supervision by the lay instructor. Other criteria being considered for final exercise selection are participants´ respective main and other impairments (cardio-pulmonary capacity, neurological symptoms, level of vision impairment and level of cognitive as well as mental capacity). The only provided equipment are low cost foam seat pads with different heights for the chair rising exercise, if necessary. The selected exercises are always repeated together with the lay instructor to ensure feasibility and safety. The exercises are defined and recorded using spreadsheets with tasks each ordered by difficulty.

**Table 2:** Training protocol (adapted from [33])

Motor abilities/ skills and exercises	Static/ Dynamic balance (a) ‘Standing’ (b) ‘Weight shifting’ (c) ‘Walking’ Strength (d) ‘Chair-rising’	
Training progression	(a) ‘Standing‘(initial position: upright bipedal stance) • With reduction of base of support: Feet shoulder-width apart ➔ side-by-side ➔ semi-tandem ➔ tandem ➔ one-legged stance • With/ without holding at table • With head movement • With eyes closed (b) ‘Weight shifting‘(initial position: upright bipedal stance) • Weight shifting sideways: With feet on the floor ➔ with raising heel of unloaded leg ➔ with lifting foot (and holding position) • Weight shifting forwards/backwards: Stride stance ➔ with raising posterior heel when shifting forwards • With/ without holding at table (c) ‘Walking’ • Small steps/ big steps/ small track width • Sideways/ backwards • From slow to fast movement velocity • With/ without double or one hand contact, with/ without walking aid (d) ‘Chair-rising’ • With booster seat if necessary (15 cm ➔ 10 cm ➔ 5 cm ➔ without booster seat) • With/ without double hand contact, with/without using armrest • From slow to fast movement velocity	
Intensity	(a) ‘Standing‘and (b) ‘Weight shifting‘ • 10–12 repetitions of10 s, including 4–6 repetitions of moderate level of difficulty and 6 repetitions of maximum level of difficulty (c) ‘Walking‘(distance 2–4 m) • 10–12 repetitions, including 7–9 repetitions of moderate level of difficulty and 3 repetitions of maximum level of difficulty (d) ‘Chair-rising’ • 5 series of 5 repetitions, including 2 series of moderate level of intensity and 3 series of higher level of intensity	
Volume	• Overall: 4-month training program • Each session (supervised): 30 min (as component of one home visit of maximally 2 h duration which further includes practicing of at least one meaningful activity and possible additional time for unstandardized interaction, e.g. conversation) • Additional self-exercise session (unsupervised or with caregiver): 10–20 min	
Frequency	• 2 supervised exercise sessions per week with a total of 35 sessions (including 32 visits with lay instructor alone, and 3 visits with lay instructor + interventionist)	• 3–4 recommended self-exercises on days without supervised exercise sessions • Frequency and time depending on patient’s capacity and caregiver’s support

In addition to the supervised program, a maximum of four exercises for further unsupervised training (static and dynamic balance exercises, chair rise) are chosen and presented with illustrated exercise sheets to the participants. For safety reasons, all balance self-exercises should be trained with both hands on a table or the back of a sturdy chair in front of the participants and a further chair directly centred closely behind so that they can sit down easily when necessary. Self-exercises are included only if it is ensured that all elements are well understood and can safely be practiced unsupervised or with support by caregivers on the days without exercises supervised by the lay instructor.

The exercise approach is based on own previous research and recent state-of-the-art research about effective, individually tailored exercise regimes for healthy older adults [33, 34], and older people with mild to moderate dementia [35, 36]. With the included exercise components, motor key performances being essential for functional recovery after hip and pelvic fractures and secondary fall prevention in older people [37–39] are addressed. Beside the aim of improving dynamic balance, an important goal of walking exercises is to improve locomotion as prerequisite for regaining independence. Additionally, prolonged gait asymmetry with an increased postural sway as result of significant strength differences between the fractured and non-fractured leg or a learned avoidance pattern might lead to a higher fall risk and should therefore be obviated as much as possible [40].

##### Visits of the lay instructors

After the initial home visit, regular home visits with exercise supervision by the lay instructor are conducted over four months with a frequency of two times per week. Each home visit takes maximally two hours including (a) 30 min for exercises, (b) practicing of at least one further physical activity according to the participant’s personal goals and (c) additional time for unstandardized interaction (e.g. conversation) comparable to usual visits provided by visitor services. All home visits and supervised exercises are recorded by the lay instructor. In accordance to previous work on peer-delivered physical activity and exercise interventions [41, 42], the authors could show in the pilot study with 17 cognitively impaired hip and pelvic fracture patients that the designed intervention approach with lay instructors is feasible and effective (Gross M, Pfeiffer K: Hip fracture and cognitive impairment: Pilot study of a post-rehabilitation exercise program, unpublished).

##### Supervision and exercise adaptations

During the 4-month exercise intervention the exercise instructor supervises the lay instructor by a minimum of five telephone calls or e-mail contacts and two further home visits.

During the home visits the intervention components (exercises, physical activity goals) are re-evaluated and adapted, if necessary. Possible fall-risk situations and barriers for physical activities like fear of falling, environmental hazards or decreased health are discussed (see below “supplementary intervention components”). Caregivers are included if possible. Besides the home visits, the exercise instructor calls the participant five times during the intervention period to receive feedback about the exercises, the lay instructor’s visits and any other issues related to the intervention.

##### Supplementary intervention components

In *Module 1* there are six supplementary components which are addressed as required during the home visits. It is intended that at least one of the components is included during each of the three home visits being accompanied by the exercise instructor. They consist of (1) addressing a minimum of three fall hazards and options for modification, (2) identifying situations in which the participant feels insecure when walking or experiences fear of falling and discussing coping strategies, (3) using walking aids safely, (4) possible self-help strategies after a fall has occurred, (5) discussing or practicing backward chaining as a strategy to get up independently from the floor, and (6) further physical activity promotion (e.g. resuming daily activities and routines, participation in community activities or local exercise classes).

Similar components have been successfully implemented in another multicomponent home-based intervention for hip fracture patients [37].

#### Description of module 2 (long-term care counselling)

After the initial home visit in *Module 1*, the exercise instructor contacts the care counsellor via phone or email. For standardized communication, a handover report is forwarded with participant’s data and, if enrolled, those of the main caregiver. Additionally, it includes the results of the initial card-sorting procedure with the patient’s individual activity goals, unmet care needs being identified so far and other obvious issues that require care counselling.

*Module 2* starts one to four weeks after the first home visit of *Module 1* and runs parallel. It comprises one initial home visit (maximum duration 2.5 h) and up to 5 telephone calls throughout the intervention period. This module focusses on facilitating the participant’s daily routines, pleasurable activities, participation and adequate care if needed. The counselling is aimed at the participant as well as the participating main family caregiver if existing.

During the home visit individual needs and wishes from the participant’s perspective are assessed by using parts of the modified German Care Counselling Inventory [43, 44]. This assessment has been developed as instrument for a comprehensive, multidimensional and systematic counselling procedure according to the German Long-Term Care Acts (SGB XI §7a). Good content validity has been confirmed (Schäufele M, Hendlmeier I, Hoell A: Report, Part A: Content validity of the German Care Counselling Inventory, unpublished). For unmet needs a care plan is developed, documented, and monitored. If needed a case review is initiated including further persons (e.g. relatives, professional services or advice centres).

The participating caregiver receives a standardized problem-solving intervention [45] including a card sorting assessment that has been described and successfully evaluated in previous studies conducted by one of the authors (KP) [30, 46]. In addition to the counselling, the participating caregiver receives information brochures every two weeks during the intervention period. The brochures include information on care relevant issues, fall prevention, memory aids for the participants, recognizing and dealing with care recipient’s pain or depressive symptoms, recommended environmental adjustments, nutrition in old age, and how to behave in case of a fall. For their own wellbeing, participating caregivers get information about local offers for further support and health promotion (e.g. relaxation techniques). During the interventions the care counsellor is in regular communication with the exercise instructor from *Module 1* if necessary. According to the manual at least one additional contact for exchange is mandatory.

#### Applied materials

##### For participants

All participants are provided with a calendar for entering the scheduled home visits of the exercise instructor, lay instructor and assessor as well as for keeping a falls and exercise diary. Every calendar includes a page with the pictures, names and telephone numbers of the study team. Finally, the participants get a folder with changeable sheets for the recommended self-exercises and additional information sheets about walker handling, coping with situations in which they feel less confident about their balance or fearful to fall, environmental hazards and backward chaining.

For the chair-rising exercise the intervention group gets the already mentioned sturdy foam pads if necessary.

##### For lay instructors

All lay instructors receive a folder with all training contents, instructions and safety issues during their initial training. Additional individual instruction and documentation sheets with the selected exercises, the number of repetitions and pause times are provided by the exercise instructor at each common home visit.

#### Interventionists

The exercise instructors in *Module 1* are physiotherapists and sports scientists, called exercise instructors, who are experienced in rehabilitation of cognitively impaired older adults. After an initial training, they are supervised by a clinical psychologist (KP) or a senior sports scientist (KH) both with long-standing experience in fall prevention, exercise programs and rehabilitation.

The interventionists in *Module 2* are a social worker and a gerontologist both highly experienced in counselling for older adults and family caregivers. Like the therapists in *Module 1* they received an initial training and are supervised by clinical psychologists (KP, MS) during the intervention period of the study.

The lay instructors have been recruited from different sources (visitor services for people with dementia, sports clubs, municipal elderly council, local newspapers, churches, public lectures on physical activity in older age). For their engagement, they get expense allowances according to the standards of local visitor services at the two recruitment sites. Before participating in the intervention, they are trained in two 4-h group sessions with a maximum of 12 participants. Every course is held together by an exercise instructor and a gerontologist. The components of the intervention delivered by the lay instructors are presented in lectures and exercises. Key elements are the exercise protocol, basic understanding about training control and progression, motivational aspects, use of walking aids, safety issues during exercising, medical or emotional exercise termination criteria and important aspects in the interaction with cognitively impaired persons.

### Control group

Participants allocated to the control group have usual access to standard care. In addition, they get the calendar for assessment dates as well as the extra sheet as memory aid showing the pictures, names and telephone numbers of the assessors.

### Outcome measures

Assessments were carried out at 4 measurement time points: -T1 (end of inpatient rehab, only participant), T1 (pre-intervention at participant’s home: week two to six post-discharge), T2 (post-intervention at participant’s home: 4 months after T1) and T3 (follow-up at participant’s home: 3 months after T2). The assessment at -T1 was performed to evaluate the further recovery process between rehabilitation and pre-intervention assessment (T1) and to identify predictors for short-term care and nursing home transition. Because of very short-term decisions on reimbursement of costs for extra rehabilitation time (usually one week or some days of ambulant rehabilitation) added to the 3-week standard rehabilitation regimen it was not possible to deliver the assessment in all cases within a narrow timeframe before discharge. Otherwise these patients have had to be excluded or assessed twice within a week.

Table 3 (participant) and Table 4 (participating caregiver) are outlined according to the SPIRIT Statement [47] and provide an overview of the assessments being performed at the defined measurement time points.

**Table 3:**
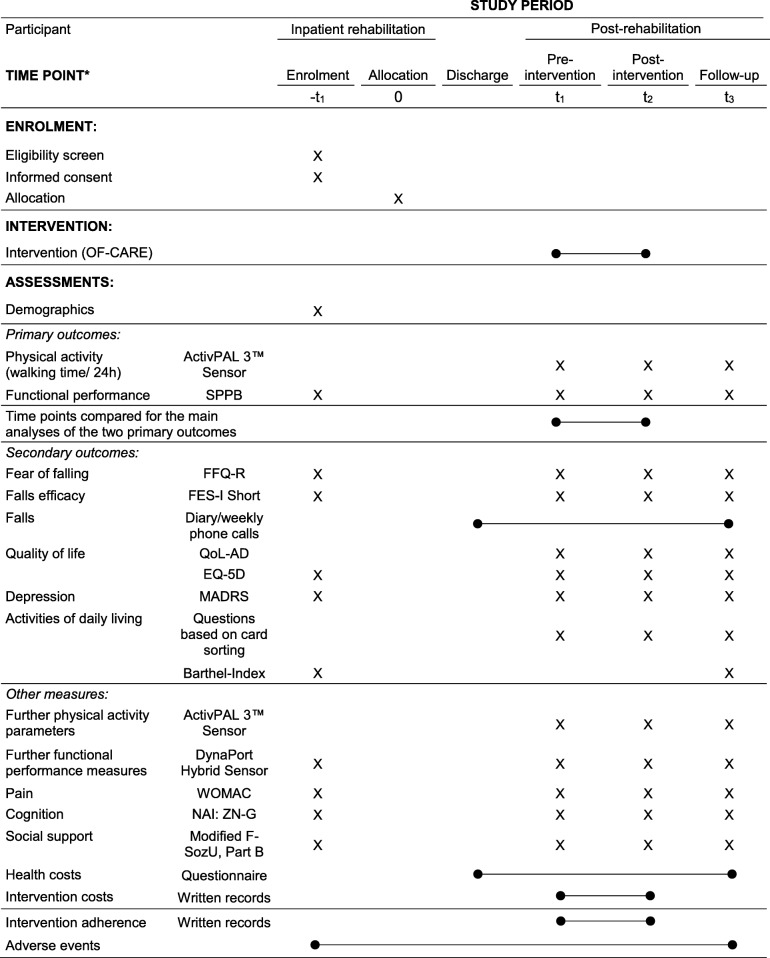
Primary and secondary outcome measures, other measures and defined measurement time points (participant, face-to-face by blinded assessors)

^*^List of specific time points in this row. EQ-5D = EuroQol- 5 Dimension; FES-I Short = Falls Efficacy Scale International – Short; FFQ-R = Fear of Falling Questionnaire – revised; F-SozU = Modified version of Social Support Questionnaire; MADRS = Montgomery–Åsberg Depression Scale; NAI: ZN-G = Nuremberg Age Inventory / subscale “repeating numbers” (both forward and backward); QoL-AD = Quality of Life in Alzheimer’s Disease; SPPB = Short Physical Performance Battery WOMAC = Western Ontario and McMaster Universities Osteoarthritis Index / subscale pain

**Table 4:**
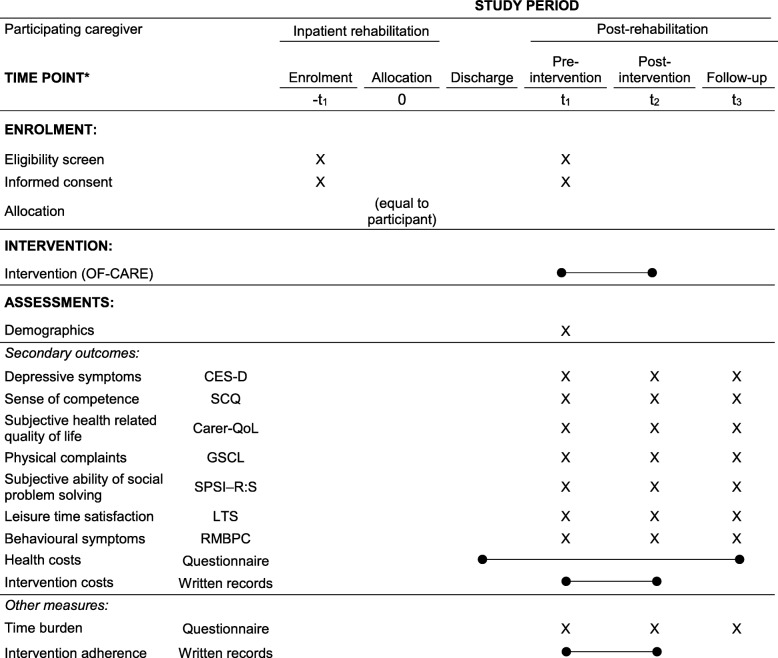
Secondary outcome measures, other measures and defined measurement time points (participating caregiver, written and, if necessary, face-to-face by blinded assessors)

^*^List of specific time points in this row. Carer-QoL = Carer-related Quality of Life questionnaire; CES-D = Centre for Epidemiological Studies – Depression Scale; GSCL = Giessen Subjective Complaints List / Subscale Pain in Limbs; LTS = Leisure Time Satisfaction Measure; RMBPC = Revised Memory and Behaviour Problems Checklist/ subscale frequency; SCQ = Sense of Competence Questionnaire – subscale 2: Satisfaction with one’s own performance as a caregiver; SPSI-R:S = Social Problem-Solving Inventory – revised / subscale: Negative problem orientation; Time burden questionnaire = Including three dimensions of care (1. body care, nutrition, mobility 2. household help IADLs (e.g. housekeeping) 3. additional supervision

It is expected that the multifactorial intervention will have a beneficial impact on participants’ physical activity and functional performance between T1 (pre-intervention) and T2 (post-intervention). Consequently, the primary outcomes include measures of these two domains. It is also expected that participants will report increases in fall related self-efficacy, quality of life, activities of daily living addressed with the assessment within intervention *module 1* and decreases in fear of falling and depressive symptoms. Compared to the control condition, no increases in fall rates are wanted in the intervention group.

The assessments are performed by trained physiotherapists and sport scientists in the participant’s home. All of them are experienced in assessing older, cognitively impaired patients, trained and supervised by the coordinating investigators of the two recruitment sites (KP, KH) and blind to the treatment condition.

#### Primary outcome measures

##### Physical activity

The daily walking duration (24 h) as proxy for physical activity is monitored with a thigh-worn inertial sensor for three consecutive weekdays (activPAL3™, PAL Technologies Ltd., Glasgow, UK) that was shown to be reliable and sufficient to predict habitual physical activity in older adults [48, 49].

##### Functional performance

Function of lower extremities is measured by the Short Physical Performance Battery (SPPB). The test is conducted according to the original protocol [50] and consists of three functional subtests assessing static balance (side-by-side-, semi-tandem-, tandem-stance), strength of the lower extremities (chair rising) and 2,44-m gait speed. It is suggested as a valid and reliable outcome measure for physical performance in community-dwelling older adults [51]. The sum-score of all subtests (ranging from 0 to 12) is used for the primary outcome analyses.

#### Secondary outcome measures

##### Fear of falling

The 6-item Fear of Falling Questionnaire revised (FFQ-R) is used for assessing fear of falling [52]. Each item is rated on a Likert-type scale from 1 (strongly disagree) to 4 (strongly agree). The total possible score ranges from 6 to 24, with higher scores indicating greater fear of falling. Previous research could demonstrate preliminary evidence that the FFQ-R is a reliable and valid instrument for assessing fear of falling in older hip-fracture patients [52]. The original questionnaire was translated into German (forward and backward). Avoidance of activities due to fear of falling is examined with a single-item question.

##### Fall-related self-efficacy

The Short Falls Efficacy Scale-International (Short FES-I) provides information of level of concern about falls for seven activities of daily living [53, 54]. The FES-I was suggested to be a valid tool for assessing also patients with cognitive impairment (MMSE sum score of validation sample: Mean = 20.9; SD = 2.1) [55].

##### Falls

Falls are recorded by implementing falls calendars and weekly telephone calls by the assessment team. This combination was found to be of acceptable accuracy in patients being in a mild to moderate stage of dementia [56]. Patients (and their caregivers) are asked to keep the diary up-to-date and fill it in each day. A fall is defined as “an unexpected event in which the patient comes to rest on the ground, floor, or lower level” [57].

##### Quality of life

The Quality Of Life In Alzheimer’s Disease questionnaire (QOL-AD) is a 13-item scale with questions on physical health, mental health, social and financial domains and an overall QOL rating [58]. The QOL-AD was found to be a reliable and valid self-report measure [59, 60]. In addition to the QOL-AD, the validated German version of the EQ-5D™ is used for health economic evaluation [61, 62].

##### Depression

Depressive symptoms are measured with the validated German version of the Montgomery-Åsberg Depression Rating Scale (MADRS) [63, 64]. The scale consists of 10 items, which can be scored from 0 to 6 during a structured interview and is recommended for assessing depressive symptoms in patients with cognitive impairment [65].

##### Activities of daily living

The Barthel Index (BI) consists of 10 items that measure a person’s daily functioning, particularly the activities of daily living and mobility. The items are rated according the Hamburg Classification Manual [66]. In addition, a self-developed questionnaire with 18 items is used to measure the instrumental activities of daily living (IADL) that are displayed on the cards which are used for patient-centred goal-setting (*Module 1*).

#### Other measures

##### Daily activity profiles

For further analysis additional dimensions (e.g. average daily number of steps, number of walking bouts, daily upright duration, daily number of sit-to-stand transfers) are extracted from the activPAL3™ monitoring data.

##### Functional performance

As supplementary measurements for functional performance, kinematic movement parameters are collected by an accelerometer (DynaPort Hybrid, McRoberts, The Hague, The Netherlands) worn at the lower back during the SPPB test. Sway parameters (sway area, sway path) are measured during the static balance subtest, sit-to-stand analysis is added by assessing angular velocity and duration of the fastest sit-to-stand sequence during the five-chair rising subtest [67]. If the patient is unable to rise from a chair (score for chair-rising test = 0), a standardized adaptation of seat height (which allows all patients to stand up without using hands) is allowed for the additional sensor-based sit-to-stand subtest. The selected adaptation is used for all further assessments to allow a comparison of the measures.

##### Pain

Pain intensity is evaluated with the German version of the Western Ontario and McMaster Universities Osteoarthritis –Scale, subscale “pain” (WOMAC) [68, 69].

##### Cognitive performance

Span of memory and working memory resources are assessed with the subscale “repeating numbers” (both forward and backward) of the Nuremberg Age Inventory (NAI: ZN-G) [70].

##### Social support

People providing support in different domains (practical support, emotional support, leisure time activities, confidential issues) are assessed with a modified version of the German Social Support Questionnaire (F-SozU Part B) [71]. It is expected that social support is correlated with physical activity and quality of life [72].

##### Health and intervention costs

Economic evaluation is conducted by recording total formal and informal care costs, acute and sub-acute hospital days, rehabilitation services and total costs of the intervention. If a patient cannot provide adequate information, his caregiver is asked with the permission of the patient to respond in this domain.

##### Adherence to intervention protocol

For *Module 1* the delivery of the card sorting task and goal setting is documented. The total number and frequency of supervised home visits, telephone calls, performed exercises (balance, strength, walking, individual exercises based on goal setting) are recorded by the interventionists and lay instructors. The participants are asked to document any additional exercises in their diaries. Non-adherence is defined if at least one of the following criteria is met: (a) less than two joint home visits of the interventionist and lay instructor, or (b) less than 19 of the targeted 32 home visits by the lay instructor without interventionist. For *Module 2* the frequency and time of face-to-face and telephone contacts with the patients (and their caregivers) are documented. In counselling scenarios without caregivers non-adherence is defined only by the missing home visit.

#### Secondary outcome measures and other measures (participating caregiver)

It is assumed that less than 50% of the patients have family caregivers who meet the inclusion criteria and consent to participate in the study. In contrast to own previous caregiver intervention studies [30, 46], caregivers are included in this study also without showing significant burden, caregiving stress behaviours or depressive symptoms. Therefore, it is expected that the statistical power is not sufficient to detect statistically significant differences in caregiver outcomes between the groups. But with measuring depressive symptoms (CES-D) [73, 74], perceived caregiver competence (SCQ; subscale two: Satisfaction with one’s own performance as a caregiver) [75, 76], quality of life (CarerQoL) [77], physical complaints (GSCL-24; subscale pain in limbs) [78], social problem-solving abilities (SPSI-R:S; subscale: negative problem orientation [79] [80]), leisure time satisfaction (LTS) [81], and frequency of the patients´ behavioural symptoms (RMBPC; subscale frequency) [82], intervention effects can be compared with previous caregiver interventions. Further information on the caregiver outcome measures are published elsewhere [46].

##### Adherence to intervention protocol

The frequency and time of face-to-face and telephone contacts with the participating caregiver are documented. Non-adherence is defined if at least one of the following criteria is met: (a) no home visit/face-to-face contact or (b) total time of follow-up telephone calls less than 45 min.

### Adverse events

In this trial a serious adverse event (SAE) is defined as any harmful disease or injury, fall-related or not, that leads to hospital admission or death [83]. Furthermore, all falls which happen during assessment or intervention independent of severity are recorded as SAE as well. Any minor or temporary clinical symptoms are only recorded as adverse events if they result in considerable restricted daily activities (e.g. staying in bed) for more than one week. All SAEs are documented according to the trial protocol. SAEs within the intervention and SAEs that requires further action beyond the study protocol (e.g. involving local services because the patient is not able to organize additional support) are directly discussed with the investigators (KP, KH, MS). In addition, the SAE documentation is discussed annually with at least one member of the independent data and safety monitoring committee (DSMC).

### Sample size

For sample size calculation, a two-step method was used. First, the sample size (N) was calculated as if a t-test on the T2 scores will be carried out, then the number of subjects was multiplied by a “design factor: (1 - ρ^2^)*N” (ρ = correlation between the outcome measured at baseline and at follow-up) to produce the number of subjects required for calculating an ANCOVA [84].

The estimated effect size calculation is based on the measured daily walking time (activPAL™) in a previous hip and pelvic-fracture study (ISRCTN79191813) as well as on the following assumptions: (a) daily mean walking time (completer, control condition with standard care) at discharge (M = 35.5 Min, SD = 27.9 Min); (b) daily walking time on average four months post-discharge from rehabilitation (M = 44.9 Min, SD = 34.8 Min); (c) a correlation of ρ = 0.45 between baseline and follow-up data; (d) an intended significant improvement of daily walking time of 30% (13.5 Min) which corresponds to an effect size Cohen’s d = 0.39; (e) β = .80 (power) and (f) α = .05 for two-sided testing.

The required number of cases for a two-sided t-test for independent samples is 106 persons in intervention and control group respectively (G*Power 3.1.9.2 [85]). The adjusted number of cases for the ANCOVA with the same power as for the t-test results in 85 persons per group [design factor: (1–0.45^2^)*106].

Using the data of the previous study in the same way for the SPPB, the design factor for this other primary outcome is lower (1–.64^2^). This means that with a sample size of 85 per group smaller effects for SPPB can be detected (Cohen’s d = 0.33) compared to the daily walking time (Cohen’s d = 0.39). Based on previous studies with hip or pelvic fracture patients a drop-out rate of 22.5% until T3 is expected. Therefore, the adjusted required number of cases is calculated with 110 patients in each group.

At the time of finalizing the study protocol for publication, an adjustment of the required sample size had been made (120 participants in each group) due to a higher drop-out rate than assumed before. Several participants had to be excluded because they went to short-term care after discharge from rehabilitation and could not return home later on.

### Data analyses

Descriptive analyses will be carried out to present baseline characteristics of both groups. Treatment effects on primary and secondary endpoints at T2 (after intervention) and T3 (at follow-up) will be analysed with analyses of covariance (ANCOVA), using baseline scores at t_1_ as covariates. In addition to intent-to-treat and completer analyses, subgroup analyses are planned (participants with pelvic fracture, MMSE total scores < 24, fear of falling, significant depressive symptoms). Since intent-to-treat analyses may underestimate active intervention components, the conduction of additional analyses (e.g. complier average causal effect) depending on type, randomness and degree of non-compliance is planned [86].

## Discussion

The aging society with an increasing number of osteoporotic fractures requires effective, sustainable and economic rehabilitation regimes to limit rising costs for long-term care [1, 7, 87]. The need for a tailored rehabilitation approach to regain maximum functional independence is even more pronounced in hip and pelvic fracture patients with cognitive impairment [22]. The idea of this home-based intervention was to develop and evaluate a set of actions designed to ensure a better continuity and coordination of care after inpatient rehabilitation. Our main aim of this transitional care approach was to promote physical activity and to ensure regular exercising with appropriate intensity and continuous supervision *(Module 1)*. It has been shown that a moderate frequency of supervised home-based exercises ensures good adherence and thus improves program efficacy in frail older people [88]. The training is embedded in home visits of the lay instructor in which further valued psychosocial aspects can be addressed for most of the patients. It is assumed that this kind of reinforcement will have an additional positive effect on the acceptance and adherence of the training.

Furthermore, it is tried to ensure that the patients receive the necessary and available support to remain living in their own homes. Based on individual goals, preferences and health status, problem-solving coping is supported, and education of patient and caregiver, as well as coordination among health professionals is provided *(Module 2)*. The demand for support will differ in this target group due to the patients´ high variety of physical and cognitive capacity. Therefore, just a minimum of contacts has been standardized in the intervention protocol in this module.

To increase the likelihood of a future implementation into services the study protocol has been developed based on visit frequencies and expense allowances of existing visitor services for Alzheimer patients and the statutory care counselling according to the national social legislation (social act/ §7a SGB XI) (*Module 2*). The inclusion of professionals, lay instructors, and caregivers is in accordance with the principle of subsidiarity that should be further strengthened by favourable conditions, local structures and reimbursement models. For implementing the intervention, it must be ensured that the lay instructors are trained and supervised adequately to consider safety and security aspects (e.g. mistreatment, abuse), to avoid physical and mental overload of the cognitively impaired patients as well as of the lay instructors themselves. Further, the lay instructors should be aware of the limits of their provided non-professional assistance. The limited set of self-exercises for the unsupervised training is selected due to security aspects and can have a too low intensity for some patients.

So far, in Germany, a standardized coordination of transitional care between health professionals of rehabilitation departments, community physiotherapy and long-term care counselling does not exist for this vulnerable target group. It is expected that a shared systematic assessment of patient’s goals in combination with regular strength and balance exercises improve or at least stabilize rehabilitation outcomes.

To the authors´ knowledge this is the first study to examine the effects of a multifactorial home-based, lay-led exercise intervention combined with long-term care counselling in cognitively impaired individuals after hip and pelvic fracture. Based on the results of this study, a future translation and implementation of the intervention in collaboration with health care insurances, visitor services, and local care counselling centres is planned.

## Ethics and dissemination

The trial is designed and conducted according to the Declaration of Helsinki [89]. Capacity evaluation of the patient’s ability to give informed consent follows the recommendations of Appelbaum [90]. If a patient is unable to decide it is ensured that a legal guardian or authorized representative gets involved in the decision to participate. Patients without any support who are not capable of making decisions during inpatient rehabilitation are excluded.

Participants and lay instructors are covered by insurance while participating in the study.

To assure that the implementation of the study is consistent with Good Clinical Practice guidelines the study is monitored by the Interdisciplinary Centre for Clinical Trials Mainz (IZKS). In addition, the DSMC assesses annually the progress of the trial, the safety of the data, and the clinical efficacy endpoints and recommends to whether to continue, modify or stop the trial. The meetings and recommendations are documented.

The results of the study will be presented at scientific congresses and published in peer-reviewed academic journals.

## Abbreviations

ADL: Activities of daily living
ANCOVA: Analysis of covariance
BI: Barthel Index
Carer-Qol: Carer-Related Quality of Life Questionnaire
CES-D: Centre of Epidemiological Studies – Depression Scale
DSMC: Data and safety monitoring committee
e.g.: For example
EQ-5D: EuroQol - 5 Dimensions
FES-I-short: Falls Efficacy Scale International – short version
FFQ-R: Fear of Falling Questionnaire - revised
F-SozU: Fragebogen zur sozialen Unterstützung [Social Support Questionnaire]
GSCL: Giessen Subjective Complaints List
IADL: Instrumental activities of daily living
ISRCTN: International Standard Randomised Controlled Trial Number
LTS: Leisure Time Satisfaction Measure
MADRS: Montgomery-Asberg Depression Scale
Min: Minutes
MMSE: Mini-Mental State Examination
NAI: ZN-G: Nürnberger Altersinventar (Zahlennachsprechen) [Nuremberg Age Inventory, (Repeating numbers)]
OF-CARE: Multifactorial intervention for osteoporotic fracture patients with mild to moderate cognitive impairment
Qol-AD: Qality of Life in Alzheimers Disease
RMBPC: Revised Memory and Behaviour Problems Checklist
S: Second
SCQ: Sense of Competence Questionnaire
SGB: Social Security Code
SPPB: Short physical performance battery
SPSI-R:S: Social Problem-Solving Inventory – revised, short version
TIDierR: Template for intervention description and replication (checklist)
WOMAC: Western Ontario and McMaster Universities Osteoarthritis Index
